# Supplementary material for: Brain-wide screen of prelimbic cortex inputs reveals a functional shift during early fear memory consolidation
Source: eLife. 2022 Jul 15;11:e78542. doi: 10.7554/eLife.78542 (PMC9286739; doi:10.7554/eLife.78542)
Supplement: Figure 2—source data 2. [file elife-78542-fig2-data2.docx]

**Figure 2 – source data 2.** Abbreviations used for the brain regions

| ACB | Nucleus accumbens |
| --- | --- |
| ACC | Anterior cingulate area |
| AON | Anterior olfactory nucleus |
| AUD | Auditory area |
| BLA | Basolateral amygdalar nucleus |
| BMA | Basomedial amygdalar nucleus |
| CA1 | Ammon's horn field CA1 |
| CA2-3 | Ammon's horn field CA2-3 |
| CLA | Claustrum |
| COA | Cortical-amygdalar area |
| CP | Caudoputamen |
| DG | Dentate gyrus |
| DORpm | Thalamus polymodal association cortex related |
| DORsm | Thalamus sensory-motor cortex related |
| DP | Dorsal peduncular area |
| EC | Entorhinal area |
| ECT | Ectorhinal area |
| EP | Endopiriform nucleus |
| FRP | Frontal pole |
| FS | Fundus of striatum |
| GU | Gustatory area |
| HATA | Hippocampo-amygdalar transition area |
| HY | Hypothalamus |
| IL | Infralimbic area |
| INS | Agranular insular area |
| LA | Lateral amygdalar nucleus |
| LSX | Lateral septal complex |
| MBmot | Midbrain motor related |
| MBsen | Midbrain sensory related |
| MBsta | Midbrain behavioral state related |
| MO | Somatomotor area |
| MOB | Main olfactory bulb |
| ORB | Orbital area |
| OT | Olfactory tubercle |
| P | Pons |
| PA | Posterior amygdalar nucleus |
| PAA | Piriform amygdalar area |
| PAL | Pallidum |
| PAR | Parasubiculum |
| PERI | Perirhinal area |
| PIR | Piriform area |
| PL | Prelimbic area |
| POST | Postsubiculum |
| PRE | Presubiculum |
| ProS | Prosubiculum |
| PTLp | Posterior parietal association area |
| RSP | Retrosplenial area |
| sAMY | striatum-like amygdalar nuclei |
| SSp | Primary somatosensory area |
| SSs | Supplemental somatosensory area |
| SUB | Subiculum |
| TEa | Temporal association area |
| TR | Postpiriform transition area |
| TT | Taenia tecta |
| VIS | Visual area |
| VISC | Visceral area |
